# Gene and noncoding RNA regulation underlying photoreceptor protection: microarray study of dietary antioxidant saffron and photobiomodulation in rat retina

**Published:** 2010-09-03

**Authors:** Riccardo Natoli, Yuan Zhu, Krisztina Valter, Silvia Bisti, Janis Eells, Jonathan Stone

**Affiliations:** 1 Division of Biomedical Sciences & Biochemistry, Research School of Biology, Australian National University; Sydney, Australia; 2 ARC Centre of Excellence in Vision Science, Sydney, Australia; 3 Department of Science and Biomedical Technology, University of L’Aquila, Coppito II, Via Vetoio, L’Aquila, Italy; 4 Department of Biomedical Sciences University of Wisconsin Milwaukee, Milwaukee, WI; 5 Bosch Institute, Discipline of Physiology and Save Sight Institute, University of Sydney, Sydney, Australia

## Abstract

**Purpose:**

To identify the genes and noncoding RNAs (ncRNAs) involved in the neuroprotective actions of a dietary antioxidant (saffron) and of photobiomodulation (PBM).

**Methods:**

We used a previously published assay of photoreceptor damage, in which albino Sprague Dawley rats raised in dim cyclic illumination (12 h 5 lux, 12 h darkness) were challenged by 24 h exposure to bright (1,000 lux) light. Experimental groups were protected against light damage by pretreatment with dietary saffron (1 mg/kg/day for 21 days) or PBM (9 J/cm^2^ at the eye, daily for 5 days). RNA from one eye of four animals in each of the six experimental groups (control, light damage [LD], saffron, PBM, saffronLD, and PBMLD) was hybridized to Affymetrix rat genome ST arrays. Quantitative real-time PCR analysis of 14 selected genes was used to validate the microarray results.

**Results:**

LD caused the regulation of 175 entities (genes and ncRNAs) beyond criterion levels (p<0.05 in comparison with controls, fold-change >2). PBM pretreatment reduced the expression of 126 of these 175 LD-regulated entities below criterion; saffron pretreatment reduced the expression of 53 entities (50 in common with PBM). In addition, PBM pretreatment regulated the expression of 67 entities not regulated by LD, while saffron pretreatment regulated 122 entities not regulated by LD (48 in common with PBM). PBM and saffron, given without LD, regulated genes and ncRNAs beyond criterion levels, but in lesser numbers than during their protective action. A high proportion of the entities regulated by LD (>90%) were known genes. By contrast, ncRNAs were prominent among the entities regulated by PBM and saffron in their neuroprotective roles (73% and 62%, respectively).

**Conclusions:**

Given alone, saffron and (more prominently) PBM both regulated significant numbers of genes and ncRNAs. Given before retinal exposure to damaging light, thus while exerting their neuroprotective action, they regulated much larger numbers of entities, among which ncRNAs were prominent. Further, the downregulation of known genes and of ncRNAs was prominent in the protective actions of both neuroprotectants. These comparisons provide an overview of gene expression induced by two neuroprotectants and provide a basis for the more focused study of their mechanisms.

## Introduction

The photoreceptors (rods and cones) of mammalian retina are the most specialized, metabolically active and fragile of the nerve cells of the retina [1–3]. Photoreceptors are also the most vulnerable of retinal cells to genetic stress, induced by mutations in genes whose expression is specific to photoreceptors, and in ubiquitously expressed genes [4,5]. The breakdown of photoreceptor stability is a major element of age-related retinal disease, and therefore of age-related blindness [6].

The stress-induced death of photoreceptors is accompanied by damage to the survivors [7–9]. Both death and damage appear to be caused by oxidative stress, i.e., by the damaging effects of partially reduced forms of oxygen, often called reactive oxygen species. Absorption of light (the normal function of photoreceptor outer segments) increases oxidation of their lipids, creating morphological and functional damage as light exposure is increased [10–12]. The idea that light-induced damage is caused by oxidative stress is supported by evidence that levels of endogenous antioxidants increase following light damage [13–15], and that exogenous antioxidants are protective [15–21], for cones [22,23] as well as rods.

We have explored the neuroprotective potential of the ancient spice saffron, which shows a strong protective effect against light-induced damage of photoreceptors [24]. The stigmata of *Crocus sativus* contain powerful antioxidants (crocin, crocetin) in biologically high concentrations [25]; their multiple C=C bonds give the stigmata their color, fragrance, taste, and antioxidant potential. Their concentration in saffron may be an evolutionarily special case, as the plant is a sterile triploid bred by vegetative propagation for its fragrance, taste, color, and medicinal properties. In a recent double blind clinical trial [26], saffron (2 μg/day over 12 weeks) induced a partial but consistent recovery of the electroretinogram elicited from the macula, and of visual acuity. We have also pioneered the use of photobiomodulation (PBM) as a retinal neuroprotectant. Red to infrared (600–1,000 nm) light at low intensities promotes wound healing in skin and oral mucosa [27], and protects photoreceptors from toxin- [28], genetic- [29], and light-induced [30] damage. Furthermore, it reduces laser-induced retinal scarring. PBM delivered transcranially reduces cerebral pathology in animal models of brain damage [31–33] and in human ischemic stroke [34]. PBM acts partly by repairing mitochondrial function and upregulating oxidative phosphorylation [35]. Again, no harmful side effects have been reported at the doses used in this in vivo work (daily doses of 5 J/cm^2^ or less). To develop the understanding of these neuroprotective effects, we have used microarray techniques to identify the genes regulated by saffron and PBM in their protective actions.

## Methods

### Experimental organization

The protective potential of dietary saffron, and of PBM, was tested using a light damage assay. Animals were treated in accordance with the ARVO Statement for the Use of Animals in Ophthalmic and Vision Research, and with protocols approved by the ANU Animal Ethics Committee. Young adult Sprague Dawley rats aged P80–120 were reared in 5 lux cyclic light, and prepared in six groups. Each group comprised two males and two females.

#### Control

These animals were raised in 5 lux cyclic light, as above. They were routinely fed a vegetable (potato or rice) matrix, developed as a biodegradable packaging material, and we used the same matrix as vehicle for feeding them with saffron.

#### Saffron-exposed only

Animals were fed saffron at 1 mg/kg/day for 3 weeks. Saffron (stigmata of *Crocus sativus*, from the Abbruzzo region in Italy) was soaked in water (at 2 mg of spice/ml H_2_O) and 12 h was allowed for the major antioxidants, which are water-soluble [25], to dissolve fully. The solute was then fed to the rats by injecting a small volume into a piece of the vegetable matrix, which the animal readily ingested. The volume for each daily feed was calculated to provide the solutes from 1 mg of saffron/kg bodyweight. Tissue was collected 24 h after the last feed.

#### Photobiomodulation-exposed only

Animals were exposed to 670 nm red light from a WARP 75 source (60mW/cm^2^, Quantum Devices Inc., Barneveld, WI). Animals were handled gently over several days until they were adapted to handling. Each was then gently restrained with a towel and held under a Plexiglas platform with the head ~2.5 cm below the platform. The WARP75 device was placed on top of the platform and turned on for 3 min. This arrangement provided a fluence of 9 J/cm^2^ at the eye. The animals did not hide from or appear agitated by the red light. Animals were treated in this way once daily for 5 days at 9:00 AM. Tissue was collected 24 h after the last treatment.

#### Light-damaged only

The animals were kept individually in Plexiglas cages, with food kept on the floor of the cages and water offered from transparent containers, to ensure uniform exposure. After overnight dark adaptation, animals were exposed to bright (1,000 lux) light for 24 h, from a white fluorescent source. Exposure began and ended at 9:00 AM

#### Saffron light damaged

Animals in this group were fed saffron for 3 weeks, as above. At 9:00 AM on the last day of feeding, they were exposed to damaging light for 24 h, as above. Tissue was collected at the end of this 24 h period.

#### Photobiomodulation light damaged

Animals in this group were exposed to PBM, as above, for 5 days. Beginning at 9:00 AM on the last day of treatment, they were exposed to damaging light for 24 h, as above. Tissue was collected at the end of this 24 h period.

### Tissue collection

At the points in the protocol specified above, animals were euthanized with Lethabarb (60 mg/kg intraperitoneally). The retina from one eye of each animal was dissected free immediately, and placed in an individual tube containing RNA*later* (Ambion Biosystems, Austin, TX), and stored at 4 °C overnight. The following day, tubes were transferred to –80 °C. The fellow eye was fixed by immersion in 4% (W/V) paraformaldehyde for examination of morphology and immunohistochemistry.

Fellow eyes were marked on the superior aspect with indelible pen for future orientation, enucleated and immersion-fixed in 4% (W/V) paraformaldehyde for 3 h, washed in 1× PBS (137 mM NaCl, 2.7 mM KCl, 10 mM Na_2_HPO_4_, 2 mM KH_2_PO_4_ at pH of 7.4) thrice, then cryoprotected by immersion in 15% (W/V) sucrose overnight. Eyes were sectioned at 12 μm on a cryostat in the superior-inferior axis.

### RNA extraction and analysis

RNA was extracted and purified using previously published methods [36]. To determine the quantity and purity of the sample, RNA was analyzed on an ND-1000 spectrophotometer (Nanodrop Technologies, Wilmington, DE) and a 2100-Bioanalyzer (Agilent Technologies, Santa Clara, CA). RNA samples were used only if the A_260_/A_280_ ratio was above 1.8 and the RNA integrity number was greater than 8.5.

### Microarray analysis

To study the changes in gene expression induced in the six experimental groups, we used 18 Affymetrix (Santa Clara, CA) Rat Genome ST arrays. These microarrays contain over 700,000 twenty-five-mer oligonucleotide features representing 27,342 genes. Labeling, hybridization, washing, and scanning of the microarray were performed at the Australian Cancer Research Foundation (ACRF) Biomolecular Resource Facility at the John Curtin School of Medical Research, Australian National University, following the manufacturers’ specifications. The arrays were scanned on the Affymetrix GeneChip 3000 7G high resolution scanner and analyzed using the GeneSpring GX v10 software (Agilent Technologies) and Partek Genomic Suite 6.4 Software (Partek Inc., St. Louis, MO). The hierarchical clustering was performed using GeneSpring on the full entity list (genes plus noncoding RNA [ncRNA]) for each of the six groups. Normalization was performed using the Robust Multichip Average (RMA) algorithm and only gene expression levels with statistical significance (p<0.05) were recorded as being “present” above background levels. Genes with expression levels below this statistical threshold were considered “absent.” For the box and whisker plot, we first ran a multivariate ANOVA (ANOVA) analysis on the six groups to identify genes whose expression was significantly varied (p<0.05, fold-change >2). This yielded a list of 187 entities, from which the box and whisker plot was generated.

The Partek Genomic Suite was used to identify genes and ncRNAs whose expression differed between experimental groups, typically between one experimental group and one control group. Data in the form of a computerized version of the .DAT file (CEL) files were imported and gene expression values were derived using the RMA algorithm on the “core” metaprobe list, which represents RefSeq genes and full-length GenBank mRNAs. For each comparison between treatment and control group, two-sample Student *t* tests were used to calculate the probability P that the expression of a gene had not changed. Genes and ncRNAs whose expression was significantly changed by treatment were selected using the criteria that p<0.05 and the fold-change in expression >2. The microarray data discussed in this publication have been uploaded to the National Center for Biotechnology Information (NCBI’s) Gene Expression Omnibus [37] and are accessible through gene expression omnibus (GEO) Series accession number GSE22818.

### Quantitative polymerase chain reaction

RNA for quantitative polymerase chain reaction (qPCR) was handled in the same way as RNA extracted for the GeneChip^®^ experiments. Three biologic groups were used, with one animal in each treatment group. Superscript III and the accompanying standard protocol (Invitrogen, Carlsbad, CA) were used to convert 1 µg of retinal RNA to cDNA (cDNA). TaqMan^®^ (Applied Biosystems, Foster City, CA) Gene Expression Mastermix (Cat# 4369514) and probes (Table 1) were used to assess the validity of gene expression changes identified in the microarray experiment using a StepOne Plus qPCR machine and StepOne software v2.1 (Applied Biosystems). Assays were performed in duplicate (to account for individual sample variability) and biologic triplicate (to account for biologic variability), with fold changes determined using comparative cycle threshold (Ct; delta-delta ct). Both glyceraldehyde 3-phosphate dehydrogenase (*Gapdh*) and β-actin (*Actb*) were used as reference genes in all qPCR experiments.

**Table 1:** TaqMan Probes used for qPCR

**Name**	**Gene symbol**	**TaqMan assay ID**
angiotensinogen (serpin peptidase inhibitor, clade A, member 8)	*Agt*	Rn00593114_m1
Beta actin	*Actb (Control)*	Rn00667869_m1
carnitine O-octanoyltransferase	*Crot*	Rn00583174_m1
chemokine (C-C motif) ligand 2	*Ccl2*	Rn01456716_g1
endothelin 2	*Edn2*	Rn00561135_m1
fatty acid binding protein 5, epidermal	*Fabp5*	Rn00821817_g1
fibroblast growth factor 2	*Fgf2*	Rn00570809_m1
glyceraldehyde-3-phosphate dehydrogenase	*Gapdh (Control)*	Rn99999916_s1
glial fibrillary acidic protein	*Gfap*	Rn00566603_m1
glutathione peroxidase 3	*Gpx3*	Rn00673916_g1
heme oxygenase (decycling) 1	*Hmox1*	Rn01536933_m1
optineurin	*Optn*	Rn00595346_m1
signal transducer and activator of transcription 3	*Stat3*	Rn00562562_m1
suppressor of cytokine signaling 3	*Socs3*	Rn00585674_s1
SWI/SNF related, matrix associated, actin dependent regulator of chromatin, subfamily d, member 1	*Smarcd1*	Rn01533317_m1

Listing of all TaqMan probes used in this project including the reference genes *Gapdh* and *Beta Actin*.

### TdT-mediated dUTP nick end labeling and quantification

Cell death was assessed by the TdT-mediated dUTP nick end labeling (TUNEL) technique to identify the fragmentation of DNA characteristic of apoptotic cells, following a previously published protocol [38] but using a fluorophore, Alexa 594, to visualize the enzymatic reaction. TUNEL-labeled sections were scanned from superior to inferior edge in 1 mm steps and the number of TUNEL-positive profiles in each 1 mm of the outer nuclear layer (ONL) was recorded. The frequency of TUNEL-positive profiles per mm of ONL was averaged from at least two sections per animal, and three or four animals were analyzed for each condition. The Student *t* test was used to compare the effects of different treatment conditions.

To demonstrate cell survival, the DNA-specific dye bisbenzimide (Calbiochem, La Jolla, CA) was used. Sections were incubated in the dye, diluted 1:10,000 in 1× PBS for 2 min at room temperature.

## Results

### Saffron and photobiomodulation (PBM) reduced photoreceptor death

**Figure 1:**
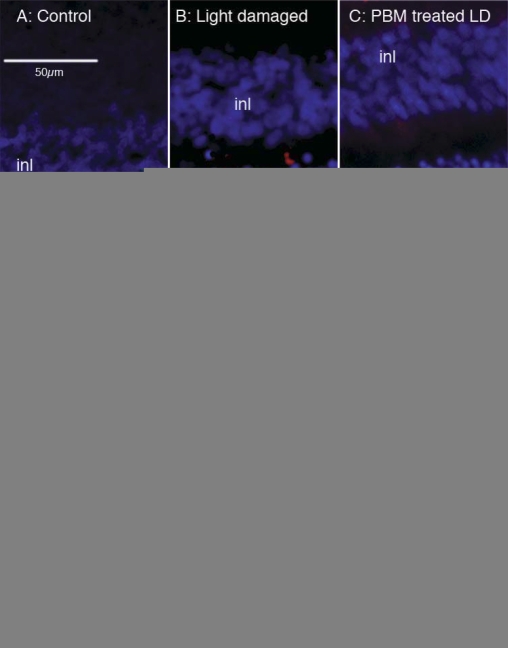
Photoreceptor rescue by saffron and photobiomodulation. Images show inner and outer nuclear layer of the retina, and the extent of damage caused by light damage to a control animal (**A**), to a animal subjected to light damage (LD; **B**) and to an animal pre-treated with photobiomodulation and then subjected to LD (**C**). The red label, applied with the TdT-mediated dUTP nick end labeling (TUNEL) technique, marks cells whose DNA is undergoing the fragmentation characteristic of apoptotic death. TUNEL-positive cells are confined to the ONL, i.e., they are the somas of photoreceptors. The number of TUNEL-positive cells is reduced by PBM pretreatment. **D**: Mean numbers of TUNEL-positive cells per mm of outer nuclear layer, for control, LD, SafLD, and PBMLD groups. The reductions in cell death caused by pretreatment with saffron and PBM were statistically significant.

Figure 1 shows the protection of light-stressed photoreceptors in rat retina achieved in the current work, confirming previous reports for saffron [24] and PBM [30]. Light stress caused the death of photoreceptors, shown as TUNEL-labeling of cells in the ONL (Figure 1B). Pretreatment with saffron or PBM reduced the number of TUNEL-positive cells in the ONL (Figure 1C, for PBM), as well as reducing the light-induced thinning of the ONL (data not shown). When quantitative data were pooled (Figure 1D), significant differences were apparent between the LD group on the one hand, and the saffron-treated and PBM-treated groups on the other (control versus LD, p<0.002 on two-tailed *t* test; LD versus saffron LD, p<0.0025; LD versus PBMLD, p<0.002).

### Global analyses of gene expression

Four approaches were used to gain an overview of entity (gene and ncRNA) expression changes in the present data.

#### Hierarchical clustering analysis

**Figure 2:**
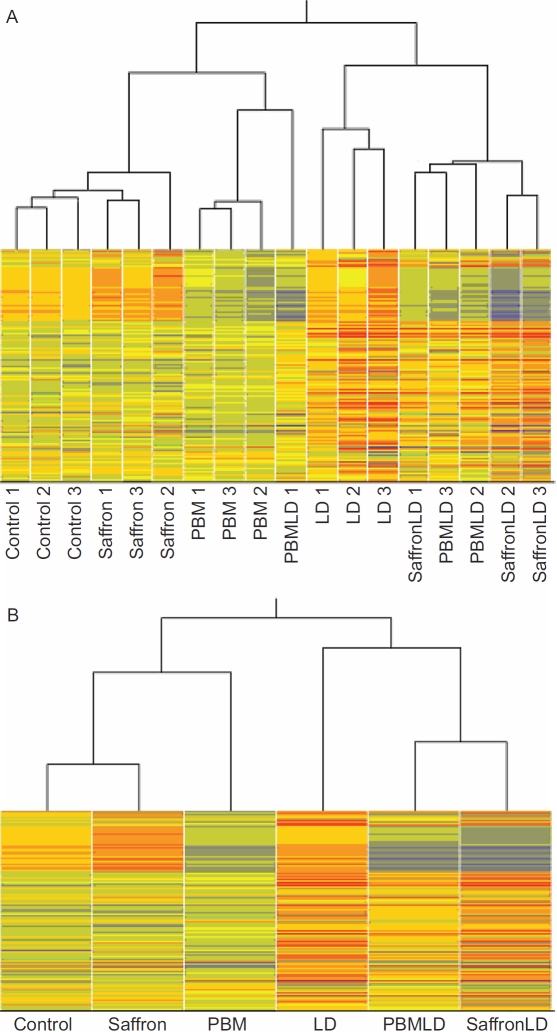
Hierarchical clustering diagram. This diagram shows the degree of similarity/difference between the 18 samples used in this study. Each column represents a sample; there were three control samples, three samples from retinas (each retina from a different animal) treated only with saffron, three from retinas/animals treated only with photobiomodulation (PBM), three from retinas/animals treated only with light damage (LD), three from retinas/animals treated with PBM and LD, and three from retinas/animals treated with saffronLD. The columns are arranged so that the most similar ones are next to each other. The branching lines at the top indicate in more detail the columns/samples that are most similar/different. **A**: With two exceptions, the three samples from each experimental group resembled each other more than samples in other experimental groups. The exceptions were PBMLD1, which resembled the PBM samples more closely than the other two PBMLD groups; saffronLD1, which resembled the PBMLD samples more closely than the other saffron LD groups. Of the three treatments used (PBM, saffron, LD), LD induced the most variable response by all assessments used. **B**: When expression values in the three samples of each of the six experimental groups were averaged, a distinct pattern of similarities emerged. The three saffron-only samples were closer to control than the PBM-only, suggesting that saffron by itself regulates fewer genes/entities than PBM. The LD-treated groups clustered together, with the two treated groups (PBMLD and SaffronLD) resembling each other more closely than the LD group. That is, treatment by PBM and Saffron before LD had broadly similar effects on the LD-induced regulation of genes/entities.

The hierarchical clustering of individual replicates (Figure 2A) indicates that the patterns of gene expression in the three samples of each group were highly reproducible. Of the 18 samples (3 samples in each of 6 groups), 16 clustered most closely with samples from the same group. One exception was PBMLD1, which clustered with the PBM samples; the other was saffronLD1 (SafLD1), which clustered with two of the PBMLD samples. Because the saffron and PBM samples clustered closely within their respective groups, the two exceptions suggest some variability in the impact of LD on gene expression.

The pattern of clustering obtained when the group replicas were averaged is shown in Figure 2B. The three samples exposed to LD cluster together, separate from the three groups not exposed, indicating that LD has a strong impact on retinal gene expression. In the three non-LD groups, the saffron-treated sample clustered closer to control retina, suggesting that PBM alone has a stronger effect on retinal gene expression than saffron alone. Within the three LD-exposed groups, the retinas also exposed to photoreceptor-protective treatment (PBMLD, SafLD1) show gene expression closer to each other than to the LD group, suggesting that PBM and saffron modify the gene expression induced by LD in broadly similar ways.

#### Distributions of gene expression in the six averaged samples—the box and whisker plot

**Figure 3:**
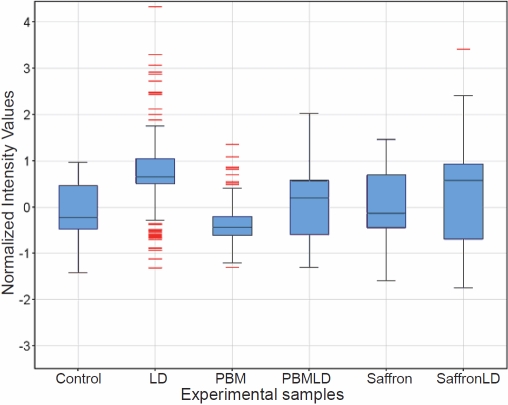
“Box and whisker” plots of the distributions of entity expression in the six experimental groups (replicates averaged). There were 187 genes included in these analyses; these were selected by a multi-ANOVA analysis of the six experimental groups (p<0.05, FC > 2). For each sample, the plot shows the median expression value of these genes as the horizontal line across the box. The upper and lower ends of the box mark the first and third quartile values, so that the box “contains” half of the sample value; the extensions show 1.5xIQR, where IQR is the interquartile range for the sample. The red lines indicate “outliers,” genes or ncRNAs whose expression level was greater or less than 1.5xIQR from the median.

An overview of gene expression in our six experimental groups is gained from the “box and whisker” plot in Figure 3. There were 187 genes included in these analyses; these were selected by a multi-ANOVA analysis of the six experimental groups (p<0.05, fold change [FC]>2).

For each sample, the plot shows the median expression value of these genes as the horizontal line across the box. The upper and lower ends of the box mark the first and third quartile values, so that the box “contains” half of the sample value; the extensions show 1.5xIQR, where IQR is the interquartile range for the sample. Expression values outside the extensions are considered outlying values, and are shown in red.

LD caused the median expression value to rise from the control value, with the expression of many entities (genes or ncRNAs) lying in outlier regions (12 above, 16 below). Saffron has relatively little effect on the distribution of gene expression levels, but PBM narrows the distribution and creates outliers. These two protective treatments thus seem to have distinctive effects. Finally, the effect of PBM and saffron given before LD was to reduce the LD-induced increase of the median and to reduce the number of outliers (to none in PBMLD, one in saffron LD).

#### Venn diagram analysis: entities associated with neuroprotection

**Figure 4:**
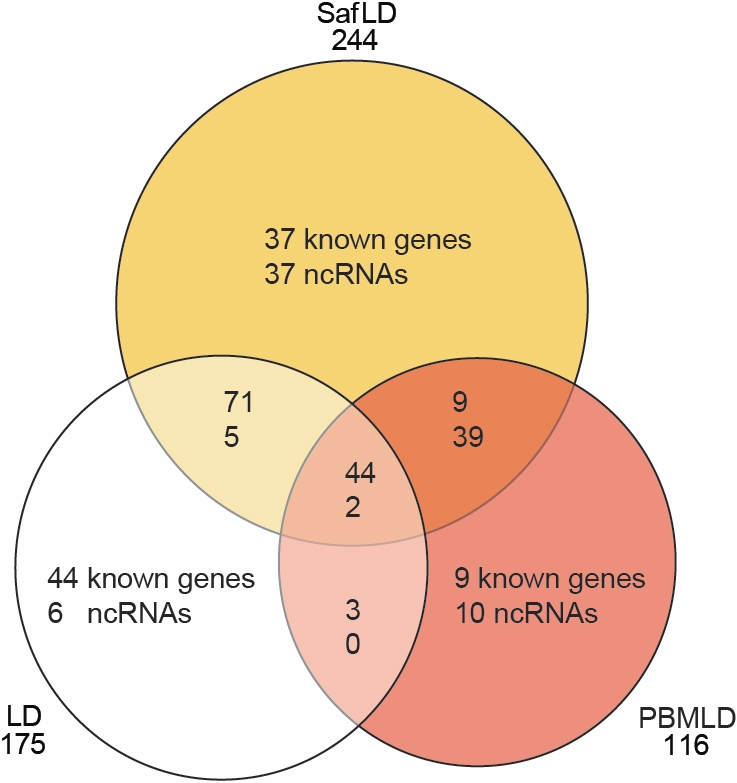
Venn Diagram showing similarity and differences between genes. The diagram is applied to three sets of regulated entities: those regulated by light damage (LD; compared to control); those regulated by LD when preceded by photobiomodulation (PBM; compared to control); and those regulated by LD when preceded by saffron feeding (compared to control). These sets were selected by two-sample Student *t* test analysis (p<0.05) and fold-change (FC>2).

A third overview of entity regulation associated with the neuroprotective actions of PBM and saffron is given by a Venn diagram analysis (Figure 4); numbers are shown separately for known genes and ncRNAs. The diagram is applied to three sets of regulated entities—those regulated by LD (compared to control); those regulated by LD when preceded by PBM (compared to control): and those regulated by LD when preceded by saffron feeding (compared to control). LD regulated 175 entities. Of these, 50 (44 known genes, 6 ncRNAs) were not regulated beyond criterion when LD was preceded by conditioning with PBM (PBMLD) or with saffron (SafLD). That is, the expression of these 50 entities (listed in Table 2) was suppressed by both PBM and saffron conditioning. Their suppression may be important in the protective actions of PBM and saffron.

**Table 2:** Genes and ncRNA significantly regulated by treatment with photobiomodulation and saffron.

**Probeset ID**	**Gene assignment**	**Gene symbol**	**RefSeq**	**p-value**	**FC (LD/C)**
10901166	angiopoietin-like 4	*Angptl4*	NM_199115	0.046613	2.20489
10738477	ADP-ribosylation factor 4-like	*Arf4l*	NM_001107052	0.027718	−2.12332
10865442	complement component 1, s subcomponent	*C1s*	NM_138900	0.027086	2.0311
10847761	Cd44 molecule	*Cd44*	NM_012924	0.017188	2.40207
10771649	chemokine (C-X-C motif) ligand 11	*Cxcl11*	NM_182952	0.041843	2.71709
10827231	cysteine-rich, angiogenic inducer, 61	*Cyr61*	NM_031327	0.024324	2.07091
10890654	estrogen receptor 2 (ER beta)	*Esr2*	NM_012754	0.009894	2.32768
10714890	Fas (TNF receptor superfamily, member 6)	*Fas*	NM_139194	0.01695	2.9447
10886031	FBJ osteosarcoma oncogene	*Fos*	NM_022197	0.008085	2.43039
10797527	growth arrest and DNA-damage-inducible, gamma	*Gadd45 g*	NM_001077640	0.01519	2.2081
10784120	gap junction protein, beta 6	*Gjb6*	NM_053388	0.033887	−2.2151
10849841	interleukin 1 beta	*Il1b*	NM_031512	0.046205	2.18761
10862908	interleukin 23 receptor	*Il23r*	XM_001067609	0.03848	2.92018
10733553	interferon regulatory factor 1	*Irf1*	NM_012591	0.005382	2.70651
10867306	hypothetical protein LOC683514	*LOC683514*	NM_001127569	0.028303	2.1278
10934056	moesin	*Msn*	NM_030863	0.031765	2.04966
10896814	myelocytomatosis oncogene	*Myc*	NM_012603	0.007411	2.7792
10920860	myeloid differentiation primary response gene 88	*Myd88*	NM_198130	0.014129	2.34728
10926588	nuclear factor of kappa light polypeptide gene enhancer i	*Nfkbie*	NM_199111	0.014731	2.17613
10750848	nuclear factor of kappa light polypeptide gene enhance	*Nfkbiz*	NM_001107095	0.00217	2.32035
10823365	purinergic receptor P2Y, G-protein coupled 12	*P2ry12*	NM_022800	0.017115	−2.38721
10792421	plasminogen activator, tissue	*Plat*	NM_013151	0.004342	2.38492
10911484	protogenin homolog (Gallus gallus)	*Prtg*	NM_001037651	0.027196	2.04521
10842475	protein tyrosine phosphatase, non-receptor type 1	*Ptpn1*	NM_012637	0.002949	2.18916
10821581	similar to hypothetical protein MGC42105	*RGD1308116*	ENSRNOT00000021964	0.013255	−2.2474
10710930	similar to hypothetical protein DKFZp434I2117	*RGD1308215*	NM_001106296	0.016796	−2.2475
10803006	similar to hypothetical protein B230399E16	*RGD1559694*	ENSRNOT00000020858	0.034301	−2.49615
10882514	RGD1560224	*RGD1560224*	ENSRNOT00000009292	0.048646	−2.31446
10855681	similar to hypothetical protein	*RGD1562590*	ENSRNOT00000015469	0.006687	2.09732
10800434	ring finger protein 125	*Rnf125*	NM_001108424	0.037824	2.3249
10893918	strawberry notch homolog 2 (Drosophila)	*Sbno2*	NM_001108068	0.007136	2.23202
10765195	selectin, platelet	*Selp*	NM_013114	0.03163	2.27171
10704505	solute carrier family 1 (neutral amino acid transporter),	*Slc1a5*	NM_175758	0.028834	2.70661
10736795	schlafen 2	*Slfn2*	NM_001107031	0.048056	2.03465
10717935	superoxide dismutase 2, mitochondrial	*Sod2*	NM_017051	0.039527	2.05701
10781273	stanniocalcin 1	*Stc1*	NM_031123	0.048406	2.26891
10869149	T-cell acute lymphocytic leukemia 2	*Tal2*	NM_001109462	0.012626	2.32825
10783880	transglutaminase 1, K polypeptide	*Tgm1*	NM_031659	0.012214	2.7564
10887306	tumor necrosis factor, alpha-induced protein 2	*Tnfaip2*	NM_001137633	0.038454	2.32289
10858967	tumor necrosis factor receptor superfamily, member 1a	*Tnfrsf1a*	NM_013091	0.013832	2.36185
10829313	transient receptor potential cation channel, subfamily	*Trpm2*	NM_001011559	0.009704	−2.04814
10802422	tubulin, beta 6	*Tubb6*	NM_001025675	0.015119	2.4974
10802995	zinc finger protein 516	*Znf516*	ENSRNOT00000021768	0.000278	2.01399
10813949	zinc finger protein 622	*Znf622*	ENSRNOT00000014423	0.011767	2.11
10821585	—		—	0.001925	−2.09732
10802710	—		—	0.005024	2.05529
10857403	—		—	0.007403	2.00653
10813885	—		—	0.00781	2.73034
10859195	—		—	0.025583	2.49356
10752799	—		—	0.026753	3.52422

Genes and ncRNAs (44 known genes, 6 ncRNAs) whose expression was significantly regulated by light damage (LD), and whose regulation was reduced below criterion when the retina was conditioned by photobiomodulation (PBM) and by saffron. These reductions in regulation may be important for the protective effects of PBM and saffron.

When saffron was given to the animal before light damage (SafLD), the expression of a large number of entities (48 in common with PBM and 74 unique to saffron) were regulated, and were not regulated by LD; i.e., their regulation can be attributed to saffron and may be important in its protective effect. Similarly, when the retina was conditioned by PBM before exposure to LD, the expressions of 67 entities (48 in common with saffron and 19 unique to PBM) was regulated, which were not regulated by LD. Their regulation can be attributed to PBM and may be important in the protective effect of PBM. The entities regulated by saffron and PBM given before LD, and not by LD, are listed in Table 3.

**Table 3:** Genes and ncRNA regulated by photobiomodulation and saffron during light damage but not regulated by light damage alone.

**Probeset ID**	**Gene_assignment**	**Gene symbol**	**RefSeq**	**p-value**	**FC (PBMLD/C)**	**FC (SafLD/C)**
10786710	biotinidase	*Btd*	NM_001012047	0.002593	−2.58996	−2.00118
10727084	cysteinyl-tRNA synthetase	*Cars*	NM_001106319	0.000472	2.04587	2.27983
10754902	discs, large homolog 1 (Drosophila)	*Dlg1*	NM_012788	0.007578	2.02011	2.20452
10814281	fatty acid binding protein 12	*Fabp12*	NM_001134614	0.03715	−2.09977	−2.09843
10797597	isoleucyl-tRNA synthetase	*Iars*	NM_001100572	0.01099	2.21568	2.14011
10796326	optineurin	*Optn*	NM_145081	0.001857	−2.04049	−2.16666
10810322	similar to calmegin	*RGD1310572*	BC097408	0.011866	2.57427	2.03946
10753017	similar to Putative protein C21orf45	*RGD1310778*	BC167102	0.000554	−2.95657	−2.95334
10758134	ubiquitin C	*Ubc*	NM_017314	0.006248	−2.2729	−2.14387
10765728	—		—	0.000316	−2.05098	−3.55913
10722459	—		—	0.000643	−4.83475	−5.7297
10722449	—		—	0.000759	−4.77591	−4.75198
10722435	—		—	0.000872	−4.53877	−3.87361
10838282	—		—	0.001154	−3.51509	−4.57167
10703224	—		—	0.001264	−3.60718	−4.66873
10722429	—		—	0.00139	−3.95495	−4.50258
10722465	—		—	0.00144	−4.49582	−5.45683
10722473	—		—	0.00149	−2.89842	−2.9913
10862359	—		—	0.00159	−4.16679	−6.62332
10894268	—		—	0.001622	−3.30485	−2.65319
10932269	—		—	0.001633	−2.51937	−3.05085
10722423	—		—	0.001744	−3.54235	−2.91368
10722461	—		—	0.002014	−3.20546	−3.46585
10722437	—		—	0.002127	−3.81721	−4.06358
10722481	—		—	0.002143	−4.18134	−4.50928
10855946	—		—	0.002276	−4.8482	−5.97919
10722433	—		—	0.002438	−2.97454	−3.52979
10722471	—		—	0.002612	−4.01264	−4.18106
10722453	—		—	0.002883	−2.32871	−2.48323
10721700	—		—	0.002935	−2.66889	−2.35149
10839872	—		—	0.003128	−3.58942	−4.13962
10722431	—		—	0.003286	−2.28992	−2.57576
10722443	—		—	0.003413	−3.36447	−3.16986
10722451	—		—	0.00356	−3.75565	−3.75383
10932228	—		—	0.003868	−2.39044	−2.32665
10722425	—		—	0.004662	−2.86663	−3.06065
10722427	—		—	0.004665	−4.23993	−4.9848
10722479	—		—	0.004887	−2.12953	−2.2299
10870039	—		—	0.005491	−2.02844	−2.04155
10722467	—		—	0.006418	−2.28354	−2.11141
10867318	—		—	0.007967	−3.77635	−2.33207
10722409	—		—	0.012757	−2.28142	−2.24389
10820008	—		—	0.014354	−2.331	−3.03034
10722383	—		—	0.017588	−2.07462	−2.12019
10722379	—		—	0.017621	−2.03053	−2.08741
10707643	—		—	0.020362	−3.27802	−3.23128
10722417	—		—	0.024253	−2.16311	−2.33892
10722387	—		—	0.028341	−2.62369	−2.64182

Genes and ncRNAs regulated by photobiomodulation (PBM) and saffron conditioning, but not by light damage (LD) alone (9 known genes, 39 ncRNAs). Their regulation by PBM and saffron conditioning suggests that they are important in the protective effects of both PBM and saffron

By separating known genes from ncRNAs, the Venn diagram analysis draws attention to the prominence of ncRNAs among the entities regulated by both saffron and PBM when they are exerting their protective actions. For example, LD regulated 175 entities, of which only 13 (7.5%) were ncRNAs. Saffron preceding LD regulated 244 entities, of which 83 (34%) were ncRNAs; while PBM preceding LD regulated 116 entities, of which 51 (44%) were ncRNAs. Among the 48 entities regulated by PBM and saffron, but not by LD, and which are therefore potentially neuroprotective entities, 39 (81%) were ncRNAs.

#### Expression changes: identified genes and noncoding RNA

**Figure 5:**
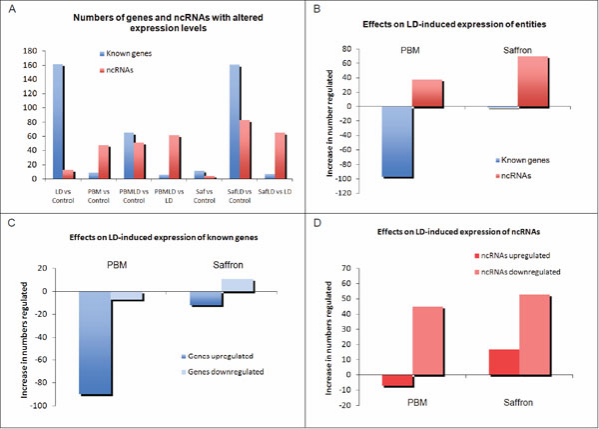
Analysis of entities regulated (known genes versus ncRNAs) and direction of regulation. **A**: Numbers of genes and ncRNAs regulated in seven comparisons among the experimental groups. SaffLD is the group given saffron before light damage (LD). **B**: Effects of saffron and photobiomodulation (PBM) on the numbers of LD-induced expression changes of known genes and ncRNAs. **C**: Direction of regulation of known genes by PBM and saffron when given as pretreatments to LD. **D**: Direction of regulation of ncRNAs by PBM and saffron when given as pretreatments to LD.

Given the prominence of ncRNAs among the entities regulated by saffron and PBM when conditioning LD, we surveyed the relative numbers of genes and ncRNAs in the seven comparisons shown in Figure 5A. As already noted, LD regulated a large number of known genes, but few ncRNAs. Conversely, ncRNAs outnumber known genes in the action of PBM on the control retina (PBM versus control); in the action of PBM when exerting its protective action against LD (PBMLD versus LD); and in the protective action of saffron (saffronLD versus LD). It seems likely that the regulation of ncRNAs accounts for a significant part of the protective effect.

This suggestion is supported by the difference comparison in Figure 5B. Measuring only changes in the numbers of genes and ncRNAs whose expression was significantly regulated by saffron or PBM before LD, the protective actions of saffron and PBM are both associated with increases in the number of ncRNAs regulated, and decreases in the numbers of identified genes whose expression was regulated.

As a final step, we considered the directions of entity expression changes in these several conditions (Figure 5C, Figure 4D). The most striking outcome of this separation is that the protective effects of PBM and saffron are associated with a decrease in the number of known genes upregulated, and an increase in the number of ncRNAs downregulated.

### Validation by real-time PCR

Thirteen genes were chosen for RT–PCR validation of the microarray outcomes; those chosen were strongly regulated and/or retina-relevant. Five genes (*Crot, Optn, Edn2, Smarcad1, Gpx3*) were significantly regulated by saffron in the LD assay. *Crot* and *smarcad1* are involved in fatty acid metabolism, *Edn2* in retinal signaling in response to injury, and *Gpx3* in antioxidative activity. *Optn* acts as an mgluR1 receptor on retinal bipolar cells. *Fabp5* is also saffron-regulated, and related to fatty acid metabolism. *Fgf* and *GFAP* are proteins upregulated by stress; *Stat3 and Socs3* are related to transduction pathways, *ccl2* to inflammatory responses, and *Agt* and heme oxygenase 1 (*Hmox1*) to cardiovascular control.

**Figure 6:**
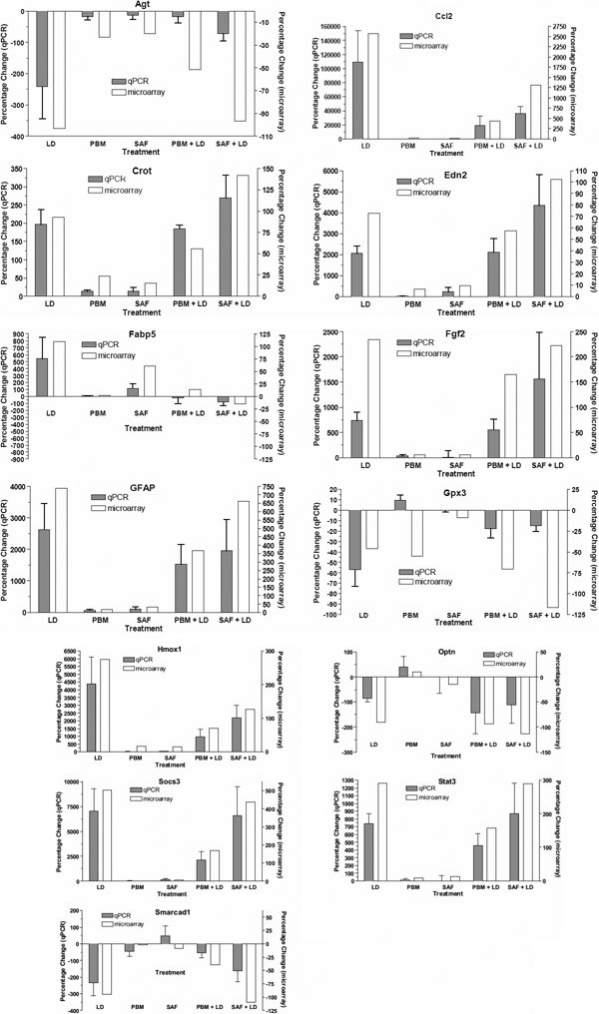
Comparisons, for thirteen selected genes, of expression changes in the six experimental groups, assessed by qPCR and microarray analysis.

Figure 6 shows a comparison for each of the 13 genes between its regulation as assessed by the microarray procedure and its regulation as assessed by RT–PCR. The correlation between the two techniques appears particularly close for *ccl2, Socs3*, *Stat2*, *Cro, Edn2, Hmox1, Fabp5,* and *smarcad.* Common trends, with quantitative differences at some sample points, are evident for *Optn, GFAP, Agt, Fgf2,* and *Gpx3*. Overall, the correlation between the two techniques seems strong.

### Entities associated with the protective actions of saffron and photobiolmodulation listed

#### Light damage–induced regulation inhibited by photobiolmodulation or saffron

The genes and ncRNAs whose regulation by LD was inhibited by PBM or saffron are listed in Table 2; as noted above, this inhibition affected principally (88%) known genes (44 known genes, 6 ncRNAs). All 50 entities were upregulated by LD; they are therefore candidates for genes and regulatory elements whose upregulation is damaging to photoreceptors.

#### Regulation by photobiolmodulation and saffron, but not LD

**Figure 7:**
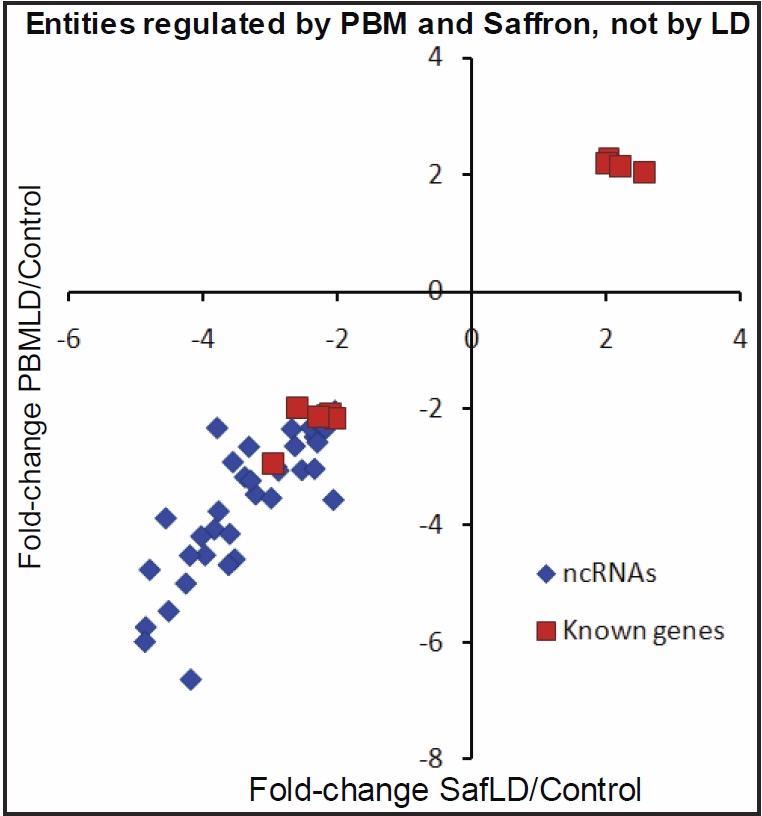
Evidence of similarity in the actions of saffron and photobiomodulation. This graph shows the correlation for 48 entities (9 known genes and 39 ncRNAs) between the change in gene expression associated with photobiomodulation (PBM) and saffron pre-treatments.

Table 3 lists genes and ncRNAs that were not regulated by LD but were regulated by PBM and saffron when conditioning (protecting) photoreceptors challenged by LD. Figure 7 shows that the effects of PBM and saffron on their regulation were highly correlated. The entity regulation shown in Table 3 contrasts in two ways with the pattern of regulation in Table 2: Most of the entities whose regulation was changed by saffron and PBM conditioning were ncRNAs (81%), and all the ncRNAs and half the known genes were downregulated.

#### Regulation by PBM or saffron, but not light damage

Further candidates for genes and ncRNAs protective to photoreceptors can be found in 74 entities (37 known genes, 37 ncRNAs) regulated by saffron (but not by PBM) when conditioning/protecting photoreceptors (Table 4), and in the 19 entities (9 known genes, 10 ncRNAs) regulated by PBM (but not by saffron) when conditioning/protecting photoreceptors (Table 5).

**Table 4:** Genes ncRNA regulated by saffron pre-conditioning but not photobiomodulation or light damage.

**Probeset ID**	**Gene_assignment**	**Gene symbol**	**RefSeq**	**p-value**	**FC (SafLD/C)**
10808041	alanyl-tRNA synthetase	Aars	NM_001100517	0.009419	2.12845
10920371	coiled-coil domain containing 72	Ccdc72	NM_001126048	0.000362	−2.15161
10753771	Cd47 molecule	Cd47	NM_019195	0.004538	2.18358
10840895	cytochrome c oxidase subunit IV isoform 2	Cox4i2	NM_053472	0.006367	−2.04762
10860548	carnitine O-octanoyltransferase	Crot	NM_031987	0.000412	2.42699
10871623	endothelin 2	Edn2	NM_012549	0.003864	2.39144
10791631	ectonucleotide pyrophosphatase/phosphodiesterase 6	Enpp6	NM_001107311	0.048871	−3.40078
10740135	fascin homolog 2, actin-bundling protein, retinal (Stro	Fscn2	NM_001107072	0.002804	2.03501
10938219	glycerol kinase	Gk	NM_024381	0.000123	2.16424
10732439	guanine nucleotide binding protein (G protein), gamma 1	Gng13	NM_001135918	0.006565	−2.23127
10733680	glutathione peroxidase 3	Gpx3	NM_022525	0.00182	−2.17933
10715200	helicase, lymphoid specific	Hells	NM_001106371	0.000882	2.2618
10863430	hexokinase 2	Hk2	NM_012735	0.029119	−2.24022
10733056	interferon gamma inducible protein 47	Ifi47	NM_172019	0.001063	2.31444
10714903	interferon-induced protein with tetratricopeptide repea	Ifit3	NM_001007694	0.004673	3.02663
10753784	intraflagellar transport 57 homolog (Chlamydomonas)	Ift57	NM_001107093	0.000101	2.67228
10815873	interleukin 12a	Il12a	NM_053390	0.003212	−2.04998
10804187	leucyl-tRNA synthetase	Lars	NM_001009637	0.000471	2.05987
10932310	mediator complex subunit 14	Med14	XM_228713	0.02603	2.23949
10923270	oligonucleotide/oligosaccharide-binding fold containin	Obfc2a	NM_001014216	8.85E-05	2.0681
10855114	olfactory receptor 820	Olr820	NM_001000974	0.025967	2.01366
10830003	pterin-4 alpha-carbinolamine dehydratase/dimerization c	Pcbd1	NM_001007601	0.009857	−2.05998
10708281	phosphodiesterase 8A	Pde8a	NM_198767	0.005571	−2.00121
10889475	peroxidasin homolog (Drosophila)	Pxdn	ENSRNOT00000060139	0.00349	−2.6691
10803138	RNA binding motif, single stranded interacting protein	Rbms2	NM_001025403	0.002005	−2.15568
10716415	similar to enolase (46.6 kDa) (2J223)	RGD1308333	NM_001134505	0.015495	2.04964
10820002	similar to Ac1147	RGD1563254	XM_577969	0.029198	−2.23707
10771190	similar to ATP-binding cassette, sub-family G (WHI	RGD1564709	NM_001107205	0.044266	2.04858
10797566	sphingosine-1-phosphate receptor 3	S1pr3	ENSRNOT00000019473	0.00326	2.25911
10750282	solute carrier family 5 (inositol transporters), member 3	Slc5a3	NM_053715	0.002179	2.1573
10842440	solute carrier family 9 (sodium/hydrogen exchanger), m	Slc9a8	NM_001025281	0.000656	2.12805
10899174	SWI/SNF related, matrix associated, actin dependent r	Smarcd1	NM_001108752	0.002766	−2.04334
10831606	transporter 1, ATP-binding cassette, sub-family B (MDR/TAP)	Tap1	NM_032055	0.011581	2.37179
10902375	TBC1 domain family, member 15	Tbc1d15	ENSRNOT00000005207	0.000945	2.0265
10858370	ubiquitin specific peptidase 18	Usp18	NM_001014058	0.019532	2.19315
10907681	zinc finger protein 385A	Zfp385a	NM_001135088	0.000479	−2.14878
10846652	zinc finger protein 385B	Zfp385b	NM_001107736	0.001897	−2.11791
10840061	—		—	0.047419	2.15179
10924441	—		—	0.044499	2.20978
10891487	—		—	0.042933	2.01488
10886190	—		—	0.040809	−2.64706
10898158	—		—	0.040237	−2.11912
10930226	—		—	0.039217	−2.1554
10915105	—		—	0.033403	−2.30406
10886854	—		—	0.032145	2.23693
10731193	—		—	0.031476	2.4581
10843907	—		—	0.030096	2.01264
10875117	—		—	0.027972	2.25392
10801781	—		—	0.026332	2.81764
10825167	—		—	0.022483	−2.8835
10722375	—		—	0.019218	−2.05422
10803001	—		—	0.018227	2.34996
10819500	—		—	0.017801	2.16908
10766880	—		—	0.015099	2.10145
10938891	—		—	0.013364	2.13311
10776604	—		—	0.012016	−2.84767
10757702	—		—	0.009955	−2.06188
10867008	—		—	0.009246	−2.29587
10891878	—		—	0.008961	−2.10309
10830454	—		—	0.008641	−2.44031
10827830	—		—	0.006903	2.30678
10897004	—		—	0.006062	2.01807
10742429	—		—	0.005794	2.98095
10834602	—		—	0.005352	−2.59778
10926624	—		—	0.005005	2.14084
10781982	—		—	0.004882	2.31978
10896630	—		—	0.003343	−2.13243
10930622	—		—	0.002504	2.29803
10756086	—		—	0.002238	2.23035
10923938	—		—	0.00188	2.22066
10938893	—		—	0.001545	2.49702
10899788	—		—	0.000365	−2.13694
10766722	—		—	8.76E-05	2.60553
10752219	—		—	1.94E-05	−2.05925

Genes and ncRNAs regulated saffron conditioning, but not by photobiomodulation (PBM) and not by light damage (LD) alone (37 known genes, 37 ncRNAs). Their regulation by saffron conditioning suggests that they are important in the protective action of saffron, and not of PBM.

**Table 5:** Genes and ncRNA regulated by photobiomodulation pre-conditioning but not saffron or light damage alone.

**Probeset ID**	**Gene_assignment**	**Gene symbol**	**RefSeq**	**p-value**	**FC (NIRLD/C)**
10916920	ATP synthase, H^+^ transporting, mitochondrial F0 complex, s	*Atp5l*	NM_212516	0.03394	−2.14869
10867731	calbindin 1	*Calb1*	NM_031984	0.03827	−2.09147
10770342	epoxide hydrolase 1, microsomal	*Ephx1*	NM_001034090	0.019435	−2.16344
10892381	nudix (nucleoside diphosphate linked moiety X)-type mo	*Nudt14*	NM_001106760	0.018954	−2.0385
10847156	olfactory receptor 673	*Olr673*	NM_001000351	0.015388	2.0757
10801260	protocadherin gamma subfamily B, 6	*Pcdhgb6*	ENSRNOT00000060466	6.03E-05	2.18939
10891322	polyglutamine-containing protein	*Pqcp*	NM_001012470	0.018634	2.00327
10796307	similar to calcium/calmodulin-dependent protein ki	*RGD1560691*	NM_001107365	0.019657	2.35325
10817552	thioredoxin interacting protein	*Txnip*	NM_001008767	0.018274	−2.32009
10886988	—		—	0.000109	25.1775
10718602	—		—	0.000392	2.10568
10721694	—		—	0.011105	−2.00257
10919224	—		—	0.011219	−2.57253
10758033	—		—	0.021858	−2.84458
10840318	—		—	0.028594	2.23714
10878967	—		—	0.031786	−2.35082
10713602	—		—	0.036424	−2.41324
10886894	—		—	0.04513	−2.03777
10797671	—		—	0.049361	−2.07756

Genes and ncRNAs regulated by photobiomodulation (PBM) conditioning, but not saffron and not by light damage (LD) alone (9 known genes, 10 ncRNAs). Their regulation by PBM conditioning suggests that they are important in the protective effects of PBM, but not in the protective action of saffron. Entities regulated by PBM, when exerting its protective action (9 known genes, 10 nc RNAs)

#### Regulation by LD, SaffronLD, and PBMLD

Genes found to be regulated by SaffronLD and LD (Table 6), PBMLD and LD (Table 7), and SaffronLD, PBMLD, and LD (Table 8) are shown in the corresponding tables. These genes are not discussed as the changes in expression levels are likely due to LD and not saffron or PBM.

**Table 6:** Genes and ncRNA regulated by saffron light damage and light damage.

**Probeset ID**	**Gene_assignment**	**Gene symbol**	**RefSeq**	**p-value**	**FC (LD/C)**	**FC (SafLD/C)**
10752839	ADAM metallopeptidase with thrombospondin type 1 motif,	*Adamts1*	NM_024400	0.003178	2.63853	2.72148
10765534	ADAM metallopeptidase with thrombospondin type 1 motif, 4	*Adamts4*	AB042272	0.005095	2.26079	2.2735
10811900	angiotensinogen (serpin peptidase inhibitor, clade A, member	*Agt*	NM_134432	0.0037	−2.29985	−2.04435
10914799	baculoviral IAP repeat-containing 3	*Birc3*	NM_023987	0.000774	3.1982	2.21198
10791652	caspase 3, apoptosis related cysteine protease	*Casp3*	NM_012922	0.001718	2.62625	2.20514
10736712	chemokine (C-C motif) ligand 12	*Ccl12*	NM_001105822	0.002288	4.38236	2.10232
10736697	chemokine (C-C motif) ligand 2	*Ccl2*	NM_031530	0.00058	38.8349	17.0997
10745677	chemokine (C-C motif) ligand 3	*Ccl3*	NM_013025	7.62E-05	19.8193	9.71154
10736863	chemokine (C-C motif) ligand 4	*Ccl4*	NM_053858	0.001026	4.06268	3.00096
10736702	chemokine (C-C motif) ligand 7	*Ccl7*	NM_001007612	0.000248	4.68541	2.67958
10729777	cholesterol 25-hydroxylase	*Ch25h*	NM_001025415	5.47E-05	4.58918	3.28027
10764069	chitinase 3-like 1	*Chi3l1*	NM_053560	0.010591	2.88704	2.22024
10912908	cytokine inducible SH2-containing protein	*Cish*	NM_031804	0.003605	10.9118	7.35678
10712853	cardiotrophin-like cytokine factor 1	*Clcf1*	NM_207615	0.009706	3.56151	2.0363
10814430	ceruloplasmin	*Cp*	NM_012532	0.003256	2.38102	2.53086
10825869	colony stimulating factor 1 (macrophage)	*Csf1*	NM_023981	0.000858	2.30889	2.15895
10775900	chemokine (C-X-C motif) ligand 1 (melanoma growth stimulat	*Cxcl1*	NM_030845	0.012601	4.11514	3.36565
10771655	chemokine (C-X-C motif) ligand 10	*Cxcl10*	NM_139089	0.003066	21.4607	7.85562
10784355	emopamil binding protein-like	*Ebpl*	NM_001108381	0.001569	−2.26226	−2.09366
10873706	Eph receptor A2	*Epha2*	NM_001108977	0.002851	2.39137	2.33589
10860231	fibrinogen-like 2	*Fgl2*	NM_053455	0.000414	4.1065	3.1288
10713045	fos-like antigen 1	*Fosl1*	NM_012953	0.000264	5.88589	4.16491
10819523	guanylate binding protein 2	*Gbp2*	NM_133624	0.000294	14.8983	7.64439
10819489	guanylate binding protein 5	*Gbp5*	NM_001108569	0.004085	7.65404	4.22902
10915843	galactosidase, beta 1-like 2	*Glb1l2*	ENSRNOT00000037790	0.002801	−3.04631	−2.17671
10806122	heme oxygenase (decycling) 1	*Hmox1*	NM_012580	0.008369	5.70686	2.9304
10908319	intercellular adhesion molecule 1	*Icam1*	NM_012967	0.00315	5.74547	3.35268
10831077	immediate early response 3	*Ier3*	NM_212505	0.005316	3.02458	2.20576
10845708	interferon induced with helicase C domain 1	*Ifih1*	NM_001109199	0.001642	3.36324	2.75239
10936365	interleukin 13 receptor, alpha 1	*Il13ra1*	NM_145789	0.004069	2.21356	2.08254
10789857	interleukin 17 receptor B	*Il17rb*	NM_001107290	0.021732	2.29043	2.26483
10813007	interleukin 6 signal transducer	*Il6st*	NM_001008725	0.018115	2.16052	2.03731
10806585	jun B proto-oncogene	*Junb*	NM_021836	0.000634	4.24227	3.86837
10844331	lipocalin 2	*Lcn2*	NM_130741	0.001852	11.4978	5.73872
10773853	leukemia inhibitory factor (cholinergic differentiation fact	*Lif*	NM_022196	0.004038	6.05907	6.27293
10751793	leucine rich repeat containing 15	*Lrrc15*	NM_145083	0.021229	3.28743	2.22908
10880293	mitogen-activated protein kinase kinase kinase 6	*Map3k6*	NM_001107909	0.018572	3.52464	2.64741
10903013	methionine-tRNA synthetase	*Mars*	NM_001127659	0.00072	2.00698	2.2033
10898561	myo-inositol oxygenase	*Miox*	NM_145771	0.002983	−2.24009	−2.20493
10815806	myeloid leukemia factor 1	*Mlf1*	NM_001107680	0.002256	2.96741	2.28383
10907881	matrix metallopeptidase 3	*Mmp3*	NM_133523	0.0008	12.1207	5.47872
10809392	metallothionein 1a	*Mt1a*	NM_138826	0.00235	3.86934	3.13553
10827989	metallothionein 2A	*Mt2A*	NM_001137564	0.002073	9.97229	5.45083
10715787	nuclear factor of kappa light polypeptide gene enhancer	*Nfkb2*	NM_001008349	0.001701	3.01772	2.0803
10727717	neuronal PAS domain protein 4	*Npas4*	NM_153626	0.002788	4.27359	2.5593
10821698	oncostatin M receptor	*Osmr*	NM_001005384	0.000642	6.0085	4.12968
10881293	podoplanin	*Pdpn*	NM_019358	0.001418	3.99897	2.61301
10930660	protein S (alpha)	*Pros1*	NM_031086	0.000526	2.09544	2.68498
10939498	RNA binding motif protein 41	*Rbm41*	NM_001109420	0.003826	2.27706	2.07025
10874929	retinol dehydrogenase 10 (all-trans)	*Rdh10*	NM_181478	0.00379	2.9943	2.32697
10900511	receptor accessory protein 6	*Reep6*	NM_001013218	8.44E-05	−3.21858	−2.57593
10883071	similar to hypothetical protein MGC38716	*RGD1304963*	ENSRNOT00000011832	0.005265	2.07828	2.02776
10816879	RGD1564171	*RGD1564171*	NM_001109186	0.003087	2.42088	2.40646
10906926	Rho family GTPase 1	*Rnd1*	NM_001013222	0.020945	2.01484	2.03486
10889399	radical S-adenosyl methionine domain containing 2	*Rsad2*	NM_138881	0.016232	12.7868	4.6819
10765173	selectin, endothelial cell	*Sele*	NM_138879	0.003772	2.68122	2.22527
10910406	sema domain, immunoglobulin domain (Ig), and GPI membr	*Sema7a*	NM_001108153	0.000297	−2.20198	−2.08698
10744687	solute carrier family 13 (sodium-dependent citrate trans	*Slc13a5*	NM_170668	0.00314	4.69584	3.06774
10805335	solute carrier family 14 (urea transporter), member 1	*Slc14a1*	NM_019346	0.003031	2.6531	2.87021
10804672	solute carrier family 26 (sulfate transporter), member 2	*Slc26a2*	NM_057127	0.015372	2.17668	2.02817
10823057	solute carrier family 7 (cationic amino acid transpor	*Slc7a11*	NM_001107673	0.000438	2.11226	2.16576
10935997	SFRS protein kinase 3	*Srpk3*	NM_184045	0.002203	2.15515	2.54938
10927842	signal transducer and activator of transcription 1	*Stat1*	NM_032612	0.000288	3.0351	2.42253
10794345	sushi domain containing 3	*Susd3*	NM_001107341	8.31E-06	−2.26654	−2.62958
10821959	threonyl-tRNA synthetase	*Tars*	NM_001006976	0.000161	2.33103	2.32162
10936482	TIMP metallopeptidase inhibitor 1	*Timp1*	NM_053819	0.002114	6.19945	3.69766
10919694	transmembrane protein 108	*Tmem108*	ENSRNOT00000014519	0.002274	−2.05022	−2.23734
10762108	transmembrane protein 116	*Tmem116*	NM_001159625	0.000345	−2.39288	−2.23365
10874198	tumor necrosis factor receptor superfamily, member 9	*Tnfrsf9*	NM_001025773	0.001067	4.29073	3.16758
10774171	uridine phosphorylase 1	*Upp1*	NM_001030025	0.001233	3.84779	2.06033
10720215	zinc finger protein 36	*Zfp36*	NM_133290	0.00113	4.38522	3.47823
10935061	—		—	0.000426	2.08993	2.27601
10766724	—		—	0.001338	3.06184	3.89387
10815496	—		—	0.003018	2.12199	2.05157
10802706	—		—	0.004766	2.01494	2.94154
10937867	—		—	0.006416	2.30821	2.21384

Genes and ncRNA regulated by both Saffron light damage (LD) and LD when compared to control.. The change in expression indicates that these genes (76 genes in total including 71 coding and 5 noncoding RNAs) change in response to light damage and not the treatment paradigm.

**Table 7:** Genes and ncRNA regulated by photobiomodulation light damage and light damage.

**Probeset ID**	**Gene_assignment**	**Gene symbol**	**RefSeq**	**p-value**	**FC (LD/C)**	**FC (PBMLD/C)**
10855701	aquaporin 1	Aqp1	NM_012778	0.000296	−2.4813	−2.26193
10761128	heat shock protein 1	Hspb1	NM_031970	0.040669	5.7848	2.67409
10834613	RGD1307355	RGD1307355	NM_001107822	0.008949	2.02622	2.00283

Genes regulated by both photobiomodulation (PBM) light damage (PBMLD) and LD when compared to control. The change in gene expressions indicate that these genes (3 genes in total) change in response to light damage and not the treatment paradigm.

**Table 8:** Genes and ncRNA regulated by all groups exposed to light damage.

**Probeset ID**	**gene_assignment**	**Gene Symbol**	**RefSeq**	**p-value**	**FC (LD/C)**	**FC (PBMLD/C)**	**FC (SafLD/C)**
10889660	aryl hydrocarbon receptor	*Ahr*	NM_013149	0.000824	3.58244	2.55846	3.37031
10860951	asparagine synthetase	*Asns*	NM_013079	0.000175	3.65332	3.59864	3.94323
10770710	activating transcription factor 3	*Atf3*	NM_012912	0.000312	14.6342	7.50088	12.4135
10906024	ceramide kinase	*Cerk*	NM_001134861	0.002068	−2.29855	−2.22234	−2.5033
10832934	carbohydrate sulfotransferase 3	*Chst3*	NM_053408	0.001969	2.29346	4.51694	4.59235
10707832	chondroitin sulfate synthase 1	*Chsy1*	NM_001106268	0.00196	2.19289	2.26058	2.38502
10847932	DEP domain containing 7	*Depdc7*	NM_001029916	0.000836	6.30169	3.61235	5.17396
10800919	early growth response 1	*Egr1*	NM_012551	0.000563	3.67302	2.5765	3.72215
10886121	estrogen-related receptor beta	*Esrrb*	NM_001008516	0.001754	−5.15267	−3.39191	−5.11574
10815026	fibroblast growth factor 2	*Fgf2*	NM_019305	0.007852	3.69169	2.80573	3.69923
10787517	growth differentiation factor 15	*Gdf15*	NM_019216	0.001794	3.10005	2.16424	2.90564
10747948	glial fibrillary acidic protein	*Gfap*	NM_017009	0.000262	9.85794	5.10179	8.53628
10853554	guanine nucleotide binding protein (G protein), gamma 11	*Gng11*	NM_022396	0.005371	2.18311	2.27589	2.84574
10910562	GRAM domain containing 2	*Gramd2*	ENSRNOT00000036798	0.002852	−2.09097	−2.0499	−2.15488
10780433	interferon regulatory factor 9	*Irf9*	NM_001012041	0.000286	5.11681	2.54387	3.98553
10878112	Jun oncogene	*Jun*	NM_021835	9.06E-05	4.10045	3.36326	4.42475
10715078	kinesin family member 11	*Kif11*	ENSRNOT00000022555	0.001127	6.71624	2.93282	6.83808
10778179	kringle containing transmembrane protein 1	*Kremen1*	NM_053649	0.00126	3.11252	2.52286	2.76134
10869693	ladinin 1	*Lad1*	NM_001107942	0.00464	3.41587	2.87979	2.67287
10766809	laminin, beta 3	*Lamb3*	ENSRNOT00000008440	0.00104	−2.13971	−2.06332	−2.29965
10731493	lipopolysaccharide-induced TNF factor	*Litaf*	NM_001105735	0.000499	3.76351	3.31786	4.20289
10832646	similar to protocadherin 15	*LOC687745*	ENSRNOT00000000744	3.36E-05	2.16788	2.11147	2.13739
10797062	MAK10 homolog, amino-acid N-acetyltransferase subunit	*Mak10*	NM_133324	0.000456	2.14755	2.06752	2.46397
10863512	methylenetetrahydrofolate dehydrogenase (NADP+ depende	*Mthfd2*	NM_001109398	0.000137	3.72708	3.3414	4.70708
10855650	pleckstrin homology domain containing, family A (phos	*Plekha8*	NM_001109235	0.002149	3.46919	2.28802	3.33038
10935064	proteolipid protein 1	*Plp1*	NM_030990	0.000277	2.15787	2.17668	2.84846
10894100	phosphatidic acid phosphatase type 2c	*Ppap2c*	NM_139252	0.000148	−3.99548	−3.52564	−3.53511
10933559	protein phosphatase, EF-hand calcium binding domain 1	*Ppef1*	NM_001034935	0.000332	−2.01839	−2.93703	−3.60683
10719616	poliovirus receptor	*PVR*	NM_017076	0.003604	3.29581	2.1509	2.47285
10730098	pyrroline-5-carboxylate synthetase (glutamate gamma-semi	*Pycs*	NM_001108524	7.37E-07	2.22935	2.00815	2.70701
10910805	similar to c-myc promoter binding protein	*RGD1562639*	XR_009072	0.001046	2.29737	2.17402	2.70157
10779602	similar to hypothetical protein	*RGD1563070*	NM_001134541	0.000934	3.13062	3.00334	3.19235
10705553	similar to F-box only protein 27	*RGD1563982*	BC091204	0.007204	2.97912	2.13711	3.00821
10703144	ribosomal protein S6 kinase polypeptide 2	*Rps6ka2*	ENSRNOT00000017809	0.000693	2.66827	2.6238	2.72578
10886621	serine (or cysteine) peptidase inhibitor, clade A, mem	*Serpina3n*	NM_031531	0.003527	15.3578	5.40098	9.53535
10778620	solute carrier family 1 (glutamate/neutral amino acid tra	*Slc1a4*	NM_198763	0.000242	2.19034	2.4281	3.04173
10785326	solute carrier family 25, member 30	*Slc25a30*	NM_001013187	0.00073	6.93878	4.0298	7.26229
10831976	solute carrier family 26, member 8	*Slc26a8*	NM_001107614	0.016312	2.42603	2.62488	2.11542
10906608	solute carrier family 38, member 2	*Slc38a2*	NM_181090	0.000719	2.11022	2.05736	2.24878
10756393	solute carrier family 7 (cationic amino acid transporter,	*Slc7a1*	NM_013111	0.001003	2.25745	2.49319	2.63527
10749372	suppressor of cytokine signaling 3	*Socs3*	NM_053565	0.000246	8.40314	3.67692	7.52167
10747506	signal transducer and activator of transcription 3	*Stat3*	NM_012747	0.000753	4.09848	2.61028	3.97928
10773496	TNFAIP3 interacting protein 2	*Tnip2*	NM_001024771	0.000385	3.20709	2.40442	4.38234
10850775	tribbles homolog 3 (Drosophila)	*Trib3*	NM_144755	0.000162	2.59849	2.31869	2.98085
10859764	—		—	0.000806	3.363	2.50985	3.02341
10768826	—		—	0.005642	2.21021	2.02298	2.28881

Genes and ncRNA regulated by all groups light damage (LD), Saffron LD and photobiomodulation (PBM) LD when compared to control. The change in expression indicates that these genes (46 genes in total, including 44 coding and 2 noncoding RNAs) change in response to light damage and not the treatment paradigm.

## Discussion

The present results provide an overview of gene and ncRNA regulation associated with the neuroprotective actions of PBM and saffron. The analyses used were chosen partly to provide validation of the method, for example the hierarchical clustering analysis in Figure 2 and the microarray-PCR comparison in Figure 6. In addition, they allow a compare-and-contrast discussion of the possible actions of saffron and PBM.

The box-and-whisker presentation in Figure 3 suggests that PBM and saffron acting on the retina in the absence of a light challenge have distinct effects. Saffron has relatively little effect on the expression of genes by the retina, but when given as pretreatment to LD, saffron reduced the large changes in gene expression induced by LD. PBM by itself had a much more significant effect on retinal gene expression than saffron, narrowing the distribution of entity expression changes and generating many “outliers.” PBM given as pretreatment to LD reduced the gene expression caused by LD toward control levels.

The Venn diagram analysis allowed a logical separation of lists of genes and ncRNAs whose regulation appears to contribute to neuroprotection; it also draws attention to the prominence of ncRNAs (rather than known genes) among the entities regulated during the protective action of PBM and saffron.

### Possible mechanisms of protection against light damage

Our study builds upon previous work showing that there are global changes in gene expression due to LD [39–42] and that antioxidants can play a role in ameliorating this stress [15,17,43,61]. A direct example is *Hmox*1, which has been previously found to be a marker for light-induced stress in the retina and could be controlled by the antioxidant dimethylthiourea [43]. Our results also show a reduction in the expression of *Hmox*1in both the LD saffron and PBMLD treated samples. In contrast to these findings, a study by Sun and colleagues reported that overexpression of *Hmox*1 is protective to the retina [44]. This suggests that *Hmox*1 act as a marker for light-induced stress rather than playing a role in the etiology of the degeneration.

Tissue antioxidant proteins have been reported to be upregulated [13,14] or their activity increased [15] following light exposure; among others, glutathiones (Gpx1), thioredoxin-1, glutathione peroxidase, glutathione-S-transferase, and glutathione reductase have been identified in these findings. In the present study, we found Gpx3 gene expression showed a reduction in the LD animals. Both saffron and PBM mitigated the changes in gene expression following LD, suggesting that both saffron and PBM have a direct regulatory effect on tissue oxidative protection.

Another possible protective mechanism involved in saffron and PBM treatment is through the reduction of inflammation due to the downregulation of chemokine (C-C motif) ligand 2 (*ccl2*). CCL2 has been found to play an important role in inflammation by inducing leukocyte recruitment and activation [45] [46]. It has been shown to be elevated in many degenerative diseases of the central nervous system, such as multiple sclerosis [47], Alzheimer disease [48], Parkinson disease [49], and amyotrophic lateral sclerosis [50]. In the eye, *ccl2* has been shown to play a role in the development of retinal degeneration; ccl2-deficient mice develop age related macular degeneration (AMD) like symptoms [51]. Our results suggest that reducing *ccl2* levels to near control levels has a direct correlation with the amount of cell death. Further investigation into the role of *ccl-2* in LD in the retina is required.

### Different forms of neuroprotection: contrasts in entity expression

LD was used in this study as an assay of the protected/vulnerable status of photoreceptors. It is relevant to recall, however, that exposure to light also involves a neuroprotective action [52,53]. Prior light experience regulates photoreceptor vulnerability to light; both ambient light experienced over long periods and a briefer exposure to very bright light upregulate mechanisms that protect the photoreceptors from a subsequent light challenge.

Recently, we [54] drew a distinction among preconditioning pretreatments that make photoreceptors resistant to LD. The distinction was between pretreatments that damage photoreceptors (examples being light [above] or hypoxia [55]) but nevertheless protect surviving photoreceptors against subsequent stress, and pretreatments that are protective without themselves damaging photoreceptors (examples being saffron [24] and PBM [28,29]). The present results show that the regulation of entity expression associated with light is very different from that associated with a nondamaging pretreatment in at least two ways. First, light regulates principally known genes, upregulating them; by contrast, PBM and saffron regulate large numbers of ncRNAs, mainly downregulating them.

### How does saffron act?

The data provide some insight into how saffron acts to protect photoreceptors against LD in the present experiments. A simple, “direct action” hypothesis for the action of an antioxidant is that it does not interact with cells, but rather acts as a direct antioxidant, shortening the lifespan of reactive oxygen species, and reducing the damage they cause. This hypothesis would predict that saffron has little effect on retinal gene expression, and this prediction is not contradicted by the list of entities (data not shown) whose expression was regulated significantly by saffron without LD. The list is short (12 known genes, 5 ncRNAs), and only one entity (an ncRNA) was regulated more than threefold. The “direct action” hypothesis appears to be contradicted, however, by the large number of genes and ncRNAs which were significantly regulated by LD, and whose regulation was reduced significantly by saffron preconditioning (Table 2); and by the large number of genes and (especially) ncRNAs whose expression was significantly regulated by saffron when given as pretreatment to LD (Table 3 and Table 4). As already noted (Figure 5), a large proportion of the entities regulated in these two ways by saffron are ncRNAs, and further understanding of the protective action of saffron will require understanding of the roles of these sequences.

With known genes, the present data allow mechanisms of saffron-induced protection to be postulated for further study. As an example, one of the genes whose expression is upregulated specifically by saffron as part of its protective action against LD (Table 4) is *endothelin 2*. Expression of this gene is associated with the upregulation of the protective/trophic factor fibroblast growth factor-2 (FGF-2), which is known to be protective against photoreceptors [56–58]. Upstream from *endothelin 2*, leukemia inhibitory factor is known to upregulate *endothelin 2* as part of the Jak/Stat pathway [59]; leukemia inhibitory factor expression has recently been shown to be protective to photoreceptors in the rat LD model [59]. Given the number of genes/entities involved, much detailed work will be required to define the mechanisms of the saffron-induced protection of photoreceptors.

### How does photobiolmodulation act?

Previous analyses of the neuroprotective action of PBM [29,35,60] have suggested that the energy of the radiation is absorbed by the mitochondrial enzyme cytochrome oxidase, which serves the key role of sequestering oxygen from the tissue for oxidative phosphorylation pathways, and the production of adenosine-5'-triphosphate (ATP). The result includes restoration of toxin-induced loss of ATP production and increased cell viability. Several studies suggest that the absorption of PBM upregulates intracellular pathways governing the redox state of the cell (reviewed [35]).

The present results confirm that PBM, given without LD, changes retinal gene expression in a significant number of entities, and that, given as a pretreatment to LD, PBM (like saffron) changes the expression of a large numbers of entities, reducing the LD-induced regulation of many (Table 2 and Table 3) and regulating many not affected by LD (Table 5). PBM, like saffron, appears to regulate many intracellular pathways when given as a pretreatment. As with saffron, a large proportion of the entities regulated by PBM are ncRNAs, and further understanding of the protective action of saffron will require understanding to the roles of these sequences.

### Neuroprotection: multiple pathways

The present results add to the knowledge of the mechanisms by which photoreceptors, and presumably other neurons, can be protected from degeneration. The present analysis of the action of saffron suggests that its action is more than that of a direct antioxidant; rather, saffron appears to interact very significantly with gene expression. Saffron is a complex of molecules [25] that includes powerful antioxidants, as well as a range of bioactive molecules. Which of these potentially active molecules, or which combination of them, accounts for the neuroprotective action of saffron remains to be determined.

PBM seems to act through at least two pathways, by reducing inflammation and by reducing oxidative damage. Future investigation of the ncRNAs regulated by PBM and saffron could reveal further clues to their mechanism of protection.
